# Genomes to fields 2024 maize genotype by environment prediction competition

**DOI:** 10.1186/s13104-026-07629-5

**Published:** 2026-02-09

**Authors:** Qiuyue Chen, Jacob D. Washburn, Dayane Cristina Lima, Maria Cinta Romay, Joseph L. Gage, James B. Holland, Alencar Xavier, Seth C. Murray, David Ertl, Marco Lopez-Cruz, Gustavo de los Campos, Fernando M. Aguate, Timothy M. Beissinger, Martin O. Bohn, Edward S. Buckler, Jode Edwards, Sherry A. Flint-Garcia, Michael A. Gore, Candice N. Hirsch, Shawn M. Kaeppler, Aida Z. Kebede, Joseph E. Knoll, John K. McKay, Richard Minyo, Osler A. Ortez, Jonathan W. Reneau, James C. Schnable, Rajandeep S. Sekhon, Maninder P. Singh, Erin E. Sparks, Addie M. Thompson, Mitchell R. Tuinstra, Jason Wallace, Wenwei Xu, Natalia de Leon

**Affiliations:** 1 Department of Plant and Agroecosystem Sciences, University of Wisconsin-Madison, Madison, WI 53706 USA; 2 Department of Crop and Soil Sciences, North Carolina State University, Raleigh, NC 27695 USA; 3 USDA-ARS Plant Genetics Research Unit, Columbia, MO 65211 USA; 4 Wisconsin Crop Innovation Center, University of Wisconsin-Madison, Madison, WI 53706 USA; 5 Institute for Genomic Diversity, Cornell University, Ithaca, NY 14853 USA; 6 USDA-ARS Plant Science Research Unit, Raleigh, NC 27606 USA; 7 Corteva Agrisciences, 8305 NW 62nd Ave, Johnston, IA 50131 USA; 8 Department of Agronomy, Purdue University, West Lafayette, IN 47907 USA; 9 Department of Soil and Crop Sciences, Texas A&M University, College Station, TX 77843 USA; 10 Iowa Corn Promotion Board, Johnston, IA 50131 USA; 11 Departments of Epidemiology & Biostatistics and Statistics & Probability, and Institute for Quantitative Health Science and Engineering, Michigan State University, East Lansing, MI 48824 USA; 12 Departments of Epidemiology & Biostatistics, Michigan State University, East Lansing, MI 48824 USA; 13 Department of Crop Science, Center for Integrated Breeding Research, University of Göttingen, Carl-Sprengel-Weg 1, 37075 Göttingen, Germany; 14 Department of Crop Sciences, University of Illinois at Urbana-Champaign, Urbana, IL 61801 USA; 15 USDA-ARS, Ithaca, NY 14853 USA; 16 USDA ARS CICGRU, 716 Farmhouse Ln, Ames, IA 50011-1051 USA; 17 Plant Breeding and Genetics Section, School of Integrative Plant Science, Cornell University, Ithaca, NY 14853 USA; 18 Department of Agronomy and Plant Genetics, University of Minnesota, St Paul, MN 55108 USA; 19 Ottawa Research and Development Center, Agriculture and Agri-Food Canada, Ottawa, ON K1A 0C6 Canada; 20 USDA-ARS Crop Genetics and Breeding Research Unit, Tifton, GA 31793 USA; 21 Department of Agricultural Biology, Colorado State University, Fort Collins, CO 80523 USA; 22 Department of Horticulture and Crop Science, Ohio State University, Wooster, OH 44691 USA; 23 Department of Plant and Soil Sciences, University of Delaware, Newark, DE 19716 USA; 24 Department of Agronomy and Horticulture, University of Nebraska-Lincoln, Lincoln, NE 68588 USA; 25 Department of Genetics and Biochemistry, Clemson University, Clemson, SC 29634 USA; 26 Department of Plant, Soil and Microbial Sciences, Michigan State University, East Lansing, MI 48824 USA; 27 Department of Agronomy, Purdue University, West Lafayette, IN 49707 USA; 28 Department of Crop & Soil Sciences, University of Georgia, Athens, GA 30602 USA; 29 Texas A&M AgriLife Research, Texas A&M University System, Lubbock, TX 79403 USA

**Keywords:** Maize (*Zea mays* ssp. *mays*), Grain yield, GxE, Prediction

## Abstract

**Objectives:**

The genomes to fields (G2F) 2024 Maize Genotype by Environment (GxE) Prediction Competition challenged participants to develop and submit their best performing models to predict grain yield for the 2024 maize GxE project field trials, using G2F data collected from 2014 to 2023 and other publicly available data.

**Data description:**

The G2F Maize GxE Project is a collaborative effort, with all generated data made publicly available. The resource presented here includes the training and test datasets used for the G2F 2024 Maize GxE Prediction Competition. Specifically, data collected from 2014 to 2023 served as the training set to predict grain yield in the 2024 test set. The dataset comprises phenotypic, genotypic, soil, weather, and environmental covariate data, along with metadata describing environments (year-location combinations). It has been curated and lightly filtered for quality control and to ensure consistent naming across years. Competitors also had access to readme files that describe the structure and content of the datasets.

## Objective

The G2F Maize GxE Project is a collaborative effort involving researchers from diverse fields. Its primary goal is to evaluate large-scale maize hybrid performance across multiple locations in the U.S., enhancing our understanding of genotype-by-environment interactions in maize. The datasets generated by this project are among the largest publicly available resources of their kind in plants, making them highly relevant to communities across genetics, agronomy, computer science, and beyond.

The 2024 G2F Maize GxE Prediction Competition was designed to engage the broader research community in developing and testing models for grain yield prediction using extensive datasets generated by the Maize GxE Project and other publicly available data sources. The competition ran from November 15, 2024, to January 15, 2025, with training and test datasets provided to participants. Participants were tasked with predicting absolute maize grain yield (Mg ha⁻¹, adjusted to 15.5% moisture) for each hybrid in each test environment (year-location combination). The winning model was determined based on the highest average Pearson Correlation Coefficient (r) which measured the strength of the linear relationship between predicted and observed 2024 grain yield across multiple locations.

This 2024 G2F Maize GxE Prediction Competition data has been made publicly available to foster collaboration and innovation in genomic prediction and environmental modeling. It serves as a resource for researchers interested in developing novel methodologies and improving the accuracy of GxE predictions in maize.

**Table 1:** Overview of G2F 2024 maize GxE prediction competition data files

Label	Name of data file	File types (file extension)	Data repository and identifier
Data file 1	readme.txt	.txt	CyVerse (10.25739/78mn-4394) [3]
Data file 2	COMPETITION_DATA_README.docx	.docx	CyVerse (10.25739/78mn-4394) [3]
Data file 3	1_Training_Trait_Data_2014_2023.csv	.csv	CyVerse (10.25739/78mn-4394) [3]
Data file 4	2_Training_Meta_Data_2014_2023.csv	.csv	CyVerse (10.25739/78mn-4394) [3]
Data file 5	3_Training_Soil_Data_2015_2023.csv	.csv	CyVerse (10.25739/78mn-4394) [3]
Data file 6	4_Training_Weather_Data_2014_2023_full_year.csv	.csv	CyVerse (10.25739/78mn-4394) [3]
Data file 7	4_Training_Weather_Data_2014_2023_seasons_only.csv	.csv	CyVerse (10.25739/78mn-4394) [3]
Data file 8	5_Genotype_Data_All_2014_2025_Hybrids.vcf	.vcf	CyVerse (10.25739/78mn-4394) [3]
Data file 9	5_Genotype_Data_All_2014_2025_Hybrids_numerical.txt	.txt	CyVerse (10.25739/78mn-4394) [3]
Data file 10	6_Training_EC_Data_2014_2023.csv	.csv	CyVerse (10.25739/78mn-4394) [3]
Data file 11	key_inbreds_G2F_2014–2025.txt	.txt	CyVerse (10.25739/78mn-4394) [3]
Data file 12	1_Submission_Template_2024.csv	.csv	CyVerse (10.25739/78mn-4394) [3]
Data file 13	2_Testing_Meta_Data_2024.csv	.csv	CyVerse (10.25739/78mn-4394) [3]
Data file 14	3_Testing_Soil_Data_2024.csv	.csv	CyVerse (10.25739/78mn-4394) [3]
Data file 15	4_Testing_Weather_Data_2024_full_year.csv	.csv	CyVerse (10.25739/78mn-4394) [3]
Data file 16	4_Testing_Weather_Data_2024_seasons_only.csv	.csv	CyVerse (10.25739/78mn-4394) [3]
Data file 17	6_Testing_EC_Data_2024.csv	.csv	CyVerse (10.25739/78mn-4394) [3]
Data file 18	7_Testing_Observed_Values.csv	.csv	CyVerse (10.25739/78mn-4394) [3]

## Data description

The Prediction Competition dataset is publicly available via CyVerse/iPlant and consists of both training and test datasets, each structured according to the specifications outlined in Table 1.

- *Training Data*: Includes phenotypic, genotypic, soil, weather (downloaded from https://power.larc.nasa.gov/), environmental covariate data, and metadata collected from 2014 to 2023 [1]. It builds upon the 2022 Maize GxE Prediction Competition dataset [2]. This dataset was used by participants to develop and train predictive models. To summarize, the training dataset contains trait data collected from approximately 180,000 maize field plots, involving 5,205 hybrids evaluated across 272 environments (year-location combination). A total of 2,425 SNP markers are available for both the training and test sets.
- *Test Data*: Contains genotypic, soil, weather, environmental covariate data, and metadata for the 2024 locations. The test dataset involves predicting 1,063 maize hybrids across 23 test locations. A submission template was also provided, listing the environments and hybrids for which participants submitted their yield predictions. While observed 2024 grain yield data was not available to participants during the competition, it is now included to allow post-competition model validation.

## Limitations

These datasets contain missing data, which is a common challenge when working with large-scale agricultural datasets. Missing data may result from data collection limitations, measurement errors, environmental disruptions, or complete plot losses. While the data have been curated and lightly filtered for quality, much of their original structure has been preserved intentionally to reflect the challenges of working with field-based experiments. Users are encouraged to make informed decisions on data cleaning, imputation, and filtering based on their research goals.

The training and test phenotype data provided as part of this release was collected from maize hybrids generated from crosses between inbred parents, which are the typical form used in maize cultivation. This release includes projected genotype calls for hybrids. However, analyses of this dataset focused on parental inbred contributions to hybrid performance may be limited by the absence of genotype information for the parents used to generate hybrids. Additionally, for some environments where precise GPS coordinates were unavailable, approximate field locations were estimated and used. Depending on the spatial resolution required, this may impact the accuracy of environmental modeling.

## Data Availability

The data described in this Data note can be freely and openly accessed on CyVerse under [10.25739/78mn-4394] [3]. Please see Table 1 for details and links to the data.

## Abbreviations

G2F: Genomes to fields
GxE: Genotype by environment
GPS: Global Positioning System
